# Microsatellite Genotyping to Distinguish Somatic *β*-HCG Secreting Carcinoma from Epithelioid Trophoblastic Tumor

**DOI:** 10.1155/2015/971970

**Published:** 2015-04-15

**Authors:** Mary Anne Brett, Monalisa Sur, Dean Daya, Jefferson Terry, Alice Lytwyn

**Affiliations:** Department of Pathology and Molecular Medicine, McMaster University, 1280 Main Street West, Room No. HSC-1N16, Hamilton, ON, Canada L8S 4L8

## Abstract

*Objective*. Morphologically, *β*-HCG secreting somatic carcinoma can be difficult to distinguish from epithelioid trophoblastic tumors (ETT). However, their distinction is critical due to their potentially differing prognoses and choice of chemotherapy. Presence of biparental alleles in ETT can be identified with molecular testing. We describe a patient who presented with metastatic carcinoma and elevated serum *β*-HCG and contrast this to an ETT in another patient. *Data and Results*. A 32-year-old female with recent possible miscarriage presented with pulmonary emboli and was found to have an increased serum *β*-HCG, a retroduodenal mass, and multiple nodules in her lungs, liver, and para-aortic lymph nodes. Biopsy showed a *β*-HCG and p63 positive epithelioid neoplasm with otherwise noncontributory immunohistochemistry. Molecular testing for biparental alleles in repeated length polymorphisms was negative, consistent with somatic origin. The second patient was a 35-year-old pregnant female with increased serum *β*-HCG and a uterine epithelioid tumor positive for *β*-HCG. Clinical and pathologic findings were characteristic of ETT and molecular testing was not required. These 2 cases illustrate that *β*-HCG secreting tumors of different etiologies may have similar appearances, and when clinical and/or IHC findings are inconclusive, molecular testing may be useful.

## 1. Introduction

Epithelioid trophoblastic tumor (ETT) is a malignant gestational neoplasm first described as a distinct entity in 1998 by Shih and Kurman [1]. It is an exceedingly rare tumor with fewer than 100 cases reported in the literature [2]. Women diagnosed with ETT often present with unexplained vaginal bleeding, amenorrhea, and a uterine mass [2], even up to 30 years after their initial pregnancy [3]. After the placenta is delivered, placental nodules can remain embedded in the myometrium and are usually reabsorbed over time. Sometimes they can persist, develop atypical cytological features, and transform into ETT [4].

ETT can pose many diagnostic challenges due to its rarity, the young age of those affected, late presentation after pregnancy, and histological mimickers. Somatic carcinoma, both *β*-HCG secreting and nonsecreting, can be morphologically difficult to distinguish from ETT. Their distinction is critical due to differing treatment options. ETT can be surgically and medically managed with hysterectomy and chemotherapy (etoposide, methotrexate, dactinomycin, cyclophosphamide, and vincristine) [5], although those with metastatic disease tend to be less responsive to conventional chemotherapy options [6].

To correctly identify ETT, genetic testing modalities may be useful in order to determine the presence of biparental alleles. Microsatellite genotyping using repeat length polymorphisms (RLP) is a genetic test that can be used to determine similarity of tissues. RLP testing can distinguish maternal from fetal tissues by identifying paternal RLPs. This testing is valuable when the differential diagnosis includes both somatic (self) and trophoblastic (nonself) neoplasms. We describe 2 cases of *β*-HCG secreting malignancy in young women that exemplify the potential value of RLP analysis as an adjunctive test in separating somatic carcinoma from gestational trophoblastic tumor.

## 2. Material and Methods: Case Presentations

*Case  1 (β-HCG secreting carcinoma)*. A 32-year-old female, with a history of an unconfirmed miscarriage, presented to the hospital with acute onset shortness of breath. A CT scan revealed bilateral pulmonary emboli and small pulmonary nodules (1 to 4 mm). An incidental elevated serum *β*-HCG (32 mIU/mL) was discovered during admission and continued to rise abnormally to a peak level of 169 mIU/mL. Subsequently, an ultrasound of the pelvis and liver showed a nongravid uterus and multiple small liver lesions. MRI confirmed the presence of metastatic hepatic nodules, as well as mesenteric masses and para-aortic nodal disease.

**Figure 1:**
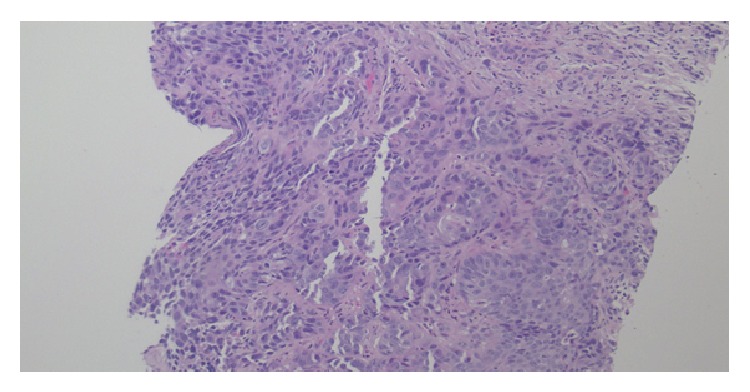
H & E. Liver core biopsy with nests of malignant epithelioid cells with eosinophilic cytoplasm.

Biopsy of the liver lesions showed nests of malignant epithelioid cells with oval to round nuclei, abundant eosinophilic cytoplasm, and extensive necrosis (Figure 1). The malignant cells showed immunohistochemical (IHC) staining that was diffusely positive for *β*-HCG and p63. It was also positive for CD10, EMA, CK7, AE1/AE3, CK5/6, p16, CA-125, and p53 (patchy). Negative stains included inhibin, PLAP, AFP, CD30, CK20, CDX2, mucin, HepPar, BRST2, ER, Napsin, TTF1, WT1, S100, HMB45, CD117, and CD34.

To determine if the malignancy was of gestational origin, a maternal blood sample and the paraffin embedded liver core biopsy containing the tumor were PCR amplified using QIAamp DNA kit (QIAGEN). Nine polymorphic microsatellite loci from 9 chromosomes, including the locus for XY determination, were amplified and analyzed. No nonmaternal (nonself) alleles were discovered in the tumor. Genetic testing supported the diagnosis of poorly differentiated carcinoma with trophoblastic differentiation.

The patient was initially started on chemotherapy with etoposide, methotrexate, and daptomycin for presumed ETT. Once the diagnosis of somatic carcinoma was made, she was switched to oxaliplatin, leucovorin, and 5-fluorouracil (FOLFOX). A repeat CT scan showed extensive worsening of disease and a new splenic lesion (1.2 cm). The patient was discharged home with palliative measures and died 3 months after her initial presentation.

*Case  2 (epithelioid trophoblastic tumor)*. A 35-year-old female (gravida 1) presented to her family physician with a positive pregnancy test. Her *β*-HCG was aberrantly elevated when compared to the expected gestational age of the fetus. Ultrasound of the pelvis showed a nonviable pregnancy, so she underwent a dilation and curettage (D&C). Despite the procedure, the patient's serum *β*-HCG level rose to 6541 mIU/mL and follow-up ultrasound showed a 1.9 cm echogenic vascular mass in the uterus. She then underwent a hysterectomy, bilateral salpingectomy, and pelvic lymph node dissection. Her serum *β*-HCG levels continually declined after the surgical management.

**Figure 2:**
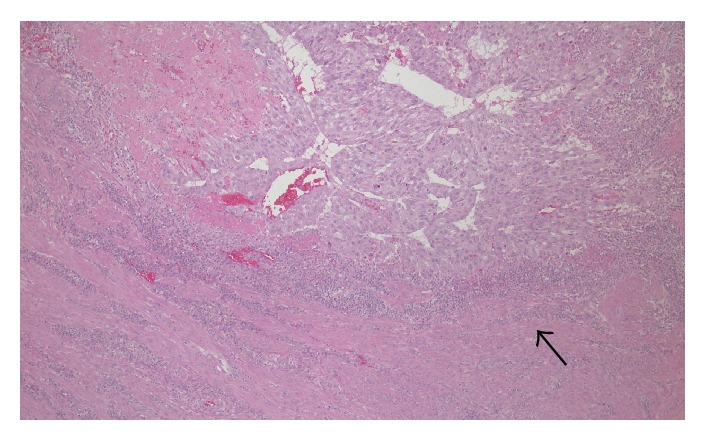
H & E. Mononucleate epithelioid tumor cells, rimmed by a lymphocytic infiltrate (arrow).

**Figure 3:**
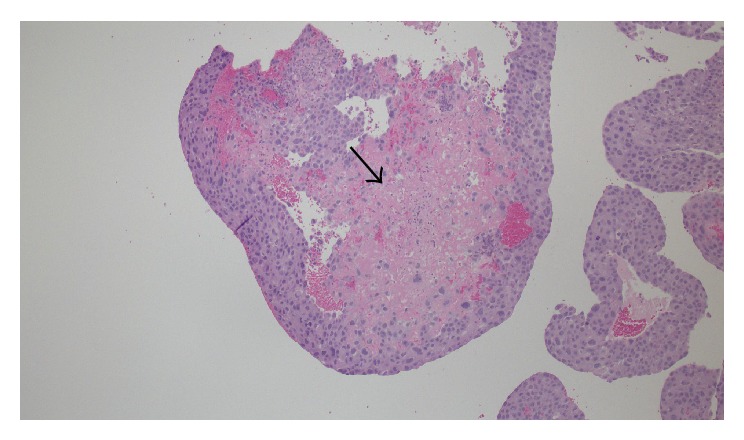
Tumor cells surrounded by eosinophilic hyaline-like material (arrow).

Pathology from the D&C and hysterectomy specimens were consistent with an epithelioid trophoblastic tumor and did not require further genetic testing. The tumor was composed of cells which showed a relatively monotonous, large, mononucleate epithelioid morphology with abundant cytoplasm within the endometrium and extending into the myometrium. The tumor had a circumscribed boarder with surrounding lymphocytic infiltrate (Figure 2). The tumor cells were surrounded by eosinophilic hyaline-like material (Figure 3) and there were areas of geographic necrosis. Tumor cells were diffusely positive for *β*-HCG, focally positive for p63, and negative for CD10, inhibin, and PLAP and had a high Ki-67 labelling index (>70%).

## 3. Results and Discussion

Gestational trophoblastic diseases are a spectrum of neoplasms that include (1) benign tumors (placental site nodule and exaggerated placental site reaction), (2) partial, complete, and invasive hydatidiform moles, and (3) malignant tumors (choriocarcinoma, epithelioid trophoblastic tumor, and placental site trophoblastic tumor).

With regard to ETT, most patients present with abnormal vaginal bleeding and are found to have a uterine or cervical primary tumor [7]. These tumors tend to respond poorly to chemotherapy and can present late, with metastases to the lungs and vagina being the most common [8]. Rare metastases have been reported in the lymph nodes, brain, liver, and skin [8]. In the literature, there are 10 cases of primary ETT presenting as an isolated lung mass without evidence of gynecological disease [7]. These lung tumors pose diagnostic challenges due to their morphological similarity to carcinoma and are easily misdiagnosed as primary nonsmall cell carcinoma (including squamous cell and pleomorphic carcinoma) or primary or metastatic germ cell tumors with trophoblastic differentiation [7]. Another issue that may hinder correct identification is that 40% of lung carcinomas aberrantly secrete *β*-HCG in the serum and stain positive on immunohistochemical analysis [3]. Histological clues to aid in diagnosing ETT within a lung biopsy are mononuclear tumor cells infiltrating alveolar spaces with preserved alveolar septa and no cytoplasmic eosinophilia or intracellular bridges [9].

There are sparse case reports and limited research to explain why ETT can present as a primary lung mass without gynecological involvement. The 2 classical etiologies include de novo transformation of trophoblastic cells that travel to the lungs during pregnancy and spontaneous regression of uterine ETT after spreading to the lungs [7]. However, the delayed presentation of lung ETT (reported up to 30 years after pregnancy) suggests that placental cells circulate through the maternal bloodstream and transform into a malignancy only after they have reached the lungs and not in the uterus after spontaneous trophoblastic tumor resolution [3].

Microsatellite genotyping is an effective test to differentiate gestational from nongestational tumors by the presence or absence of biparental alleles. This distinction is important when examining other more common trophoblastic diseases, such as gestational and nongestational choriocarcinoma. Most choriocarcinomas are derived from pregnancies, with very few arising independently from gestation (primary/nongestational choriocarcinoma) [10]. It is imperative to correctly distinguish between these tumors due to differing sensitivity to chemotherapy and overall prognosis [10]. Using microsatellite genotyping, choriocarcinoma can display 3 genetic patterns: (1) nongestational choriocarcinoma where all tumor alleles match those of the patient, (2) gestational choriocarcinoma, originating from androgenesis, where all tumor alleles belong to the partner and none are derived from the patient, and (3) gestational choriocarcinoma, originating from a diploid embryo, where the tumor alleles are of biparental origin [10]. Genetic testing in this situation would impact patient care because gestational choriocarcinomas, containing both patient and paternal genetic material, tend to be more chemosensitive and have a better prognosis than those of uniparental origin [10].

We report 2 patients who presented with elevated *β*-HCG as a result of differing neoplastic processes and how microsatellite genotyping, as an adjunct test, was helpful in reaching the final diagnosis. Epithelioid trophoblastic tumor is a very rare malignancy and therefore poses diagnostic pitfalls which must be addressed.

Several factors made our first case an especially challenging diagnosis. (1) The patient's possible history of miscarriage and young age were more in keeping with GTD as opposed to a somatic neoplasm, which would be less likely in this age group. (2) The mild incidental increase in *β*-HCG could have been due to ectopic pregnancy, GTD, or an extrauterine malignancy with trophoblastic differentiation. (3) Her presentation of nodules in the lungs, liver, mesentery, para-aortic lymph nodes, and eventually spleen, without evidence of disease in the uterus, remained unusual for GTD. Nonetheless, there are several case reports of ETT presenting in unusual locations including the lungs [5, 11], with unremarkable reproductive organs. (4) On light microscopy, the malignant cells resembled those seen in ETT, having large epithelioid cells with abundant eosinophilic cytoplasm and large areas of necrosis. (5) Multiple IHC panels were overall inconclusive therefore molecular genotyping was necessary and valuable.

In our second case, the clinical presentation, morphology of the neoplastic cells, IHC profile, and site of primary tumor were all consistent with ETT. Molecular testing with repeat length polymorphisms was not required for diagnosis.

In young female patients with elevated *β*-HCG, somatic metastatic carcinoma is unusual, so it is important to consider other diagnoses including gestational trophoblastic tumor. When the primary tumor site is unknown, histology and immunohistochemistry fail to delineate the origin, and epithelioid trophoblastic tumor remains in the differential diagnosis, then microsatellite genotyping is recommended.
